# *In vivo* virulence of *Mycobacterium tuberculosis* depends on a single homologue of the LytR-CpsA-Psr proteins

**DOI:** 10.1038/s41598-018-22012-6

**Published:** 2018-03-02

**Authors:** S. Malm, S. Maaß, U. E. Schaible, S. Ehlers, S. Niemann

**Affiliations:** 1 Molecular and Experimental Mycobacteriology, Priority Area Infections, Research Center Borstel – Leibniz Center for Medicine and Biosciences, 23845 Borstel, Germany; 2 Cellular Microbiology, Priority Area Infections, Research Center Borstel – Leibniz Center for Medicine and Biosciences, 23845 Borstel, Germany; 3 Molecular Inflammation Medicine, Priority Area Infections, Research Center Borstel – Leibniz Center for Medicine and Biosciences, 23845 Borstel, Germany; 4 German Center for Infection Research, Borstel Site, Borstel, Germany

## Abstract

LytR-cpsA-Psr (LCP) domain containing proteins fulfil important functions in bacterial cell wall synthesis. In *Mycobacterium tuberculosis* complex (*Mtbc*) strains, the causative agents of tuberculosis (TB), the genes Rv3484 and Rv3267 encode for LCP proteins which are putatively involved in arabinogalactan transfer to peptidoglycan. To evaluate the significance of Rv3484 for *Mtbc* virulence, we generated a deletion mutant in the *Mtbc* strain H37Rv and studied its survival in mice upon aerosol infection. The deletion mutant failed to establish infection demonstrating that Rv3484 is essential for growth in mice. Following an initial phase of marginal replication in the lungs until day 21, the Rv3484 deletion mutant was almost eliminated by day 180 *post-infectionem*. Interestingly, the mutant also showed higher levels of resistance to meropenem/clavulanate and lysozyme, both targeting peptidoglycan structure. We conclude that Rv3484 is essential for *Mtbc* virulence *in vivo* where its loss of function cannot be compensated by Rv3267.

## Introduction

Tuberculosis (TB) is, in conjunction with HIV infection, the leading cause of mortality and morbidity due to a single infectious agent worldwide^1^. Increasing numbers of cases with multidrug (MDR) and extensively drug (XDR) resistant *Mycobacterium tuberculosis* complex (*Mtbc*) strains complicate TB control and potentially lead to an era in which current anti-TB drugs are no longer effective^1,2^. This underscores the urgent need to identify new drug targets to design new and more effective treatment regimens. An ideal drug target is essential for bacterial survival under *in vivo* infection conditions. Several of the current antibiotics target cell wall synthesis and thereby interfere with an essential structure for bacterial cell integrity^3^.

Accordingly, we were interested in genes putatively involved in mycobacterial cell wall homeostasis. Based on the previously defined core transcriptome of 17 *Mtbc* strains responding to intracellular conditions in murine bone marrow derived macrophages (mBMDM)^4^, we selected Rv3484, which contains besides the LytR-cpsA-psr (E-value 3.49e-51) (LCP) domain conserved Csp2a (E-value 1.48e-75), PRK09379 (E-value 2.30e-16) and LytR_C (E-value 3.44e-11) domains as detected by NCBI CDD search^5^. Proteins containing these domains have been associated with bacterial cell wall metabolism and regulatory functions even though the exact mechanism remained elusive^6–13^. Phosphotransferase activity was described for LCP proteins of Gram-positive bacteria, where the transfer of anionic cell wall polymers, such as teichoic acids or capsular polysaccharides, to peptidoglycan (PGN) was attributed to LCP proteins^14,15^. Recently, *in vitro* reconstitution studies revealed ligase activity for the *Staphylococcus aureus* LCP proteins, LcpA, LcpB and LcpC. These proteins transferred wall teichoic acid precursor molecules to PGN oligomers^16^. Additional results further indicated that LCP proteins catalyze the transfer of diverse substrates to different acceptor moieties such as the transfer of the lipid-linked ManNAc-GlcNAc-GlcNAc of *Bacillus anthracis*. In *B. anthracis*, the LCP homologues, *lcpB2*, *lcpC* and *lcpD* were also able to complement for the lack of wall teichoic acid attachment to PGN in *S. aureus* LCP mutants strains^17^. In addition glycoprotein glycosylation in *Actinomyces oris* has been ascribed to an LCP protein^18^.

The cell wall core of mycobacteria and corynebacteria consists of PGN, which is covalently bound to arabinogalactan (AG) and mycolic acids. In *Mtbc* this structure builds up the scaffold for the asymmetric outer lipid bilayer, which in turn, serves as a matrix for virulence associated lipids such as phtiocerol dimycocerosates (PDIM), phenolic glycolipids (PGL) and trehalose dimycolate (TDM), as well as e.g. mannosylated lipoarabinomannan (manLAM). The cell wall in its complexity is responsible for many of the properties of the *Mtbc*, e.g. its rigidity and resistance to common disinfectants or its low permeability for antibiotics; it provides immune modulatory compounds and ligands for host cell interaction and preserves bacterial cell integrity^19–21^. Homologues of the LCP proteins have been identified in the genomes of *Corynebacterium glutamicum*, *M. marinum* and *Mtbc*^22,23^. In *M. marinum*, orthologues of all LCP proteins of *Mtbc* are present (MMAR_4858, MMAR_1274, MMAR_4966, and MMAR_5392; Rv0822c, Rv3267, Rv3484, and Rv3840, respectively). A *M. marinum* transposon mutant defective in MMAR_4966 revealed altered colony morphology, cell surface properties, lower growth rate *in vitro*, enhanced susceptibility to erythromycin, vancomycin and penicillin and altered cell wall permeability for hydrophobic compounds. Comparing cell wall compositions between wild-type and mutant *M. marinum* cells showed an imbalance in the AG/PGN-ratio. Furthermore the mutant was less virulent in the murine macrophage cell line RAW 264.7 and the zebrafish model^22^.

Grzegorzewicz *et al*. analyzed heterologously expressed Rv3484, Rv3267 and Rv0822c which showed pyrophosphatase activity, corroborating their proposed function as LCP proteins. They obtained single mutants for Rv3484 and Rv3267 in the virulent *Mtb* CDC1551 strain and on the background of an avirulent auxotroph H37Rv mutant, whereas a double mutant could only be generated for Rv0822c/Rv3267 but not for the other combinations. It was concluded that Rv3484 and Rv3267 substitute for each other’s function, however, together are essential for viability *in vitro* whereas Rv0822c plays a redundant role. Analyzing the rhamnose/GlcNAc ratio as marker for AG and PGN corroborated this hypothesis, as no significant differences were observed between both mutants and the parental wild-type strain. Furthermore, total lipids, mycolic acids and cell wall polysaccharides did not differ between mutants and wild-type mycobacteria. Notably, the Rv3267 deficient mutant exhibited enhanced sensitivity to antibiotics, which was speculated to be due to synergistic effects between Rv3267 deficiency and cell wall targeting antibiotics such as vancomycin or β-lactams. A predominant role of Rv3267 in AG attachment was suggested^23^. Catalyzing the transfer of labelled galactose containing cell wall material to peptidoglycan *in vitro* was also described for Rv3267^24^.

To further characterize the role of Rv3484 for virulence, we generated an unmarked in-frame deletion mutant of Rv3484 in *Mtb* H37Rv and investigated its survival *in vivo*. Our findings show that inactivation of Rv3484 alone is sufficient to abolish long term growth of *Mtb* upon aerosol infection in C57BL/6 mice.

## Results

### Deletion of Rv3484 of Mtb H37Rv

**Figure 1:**
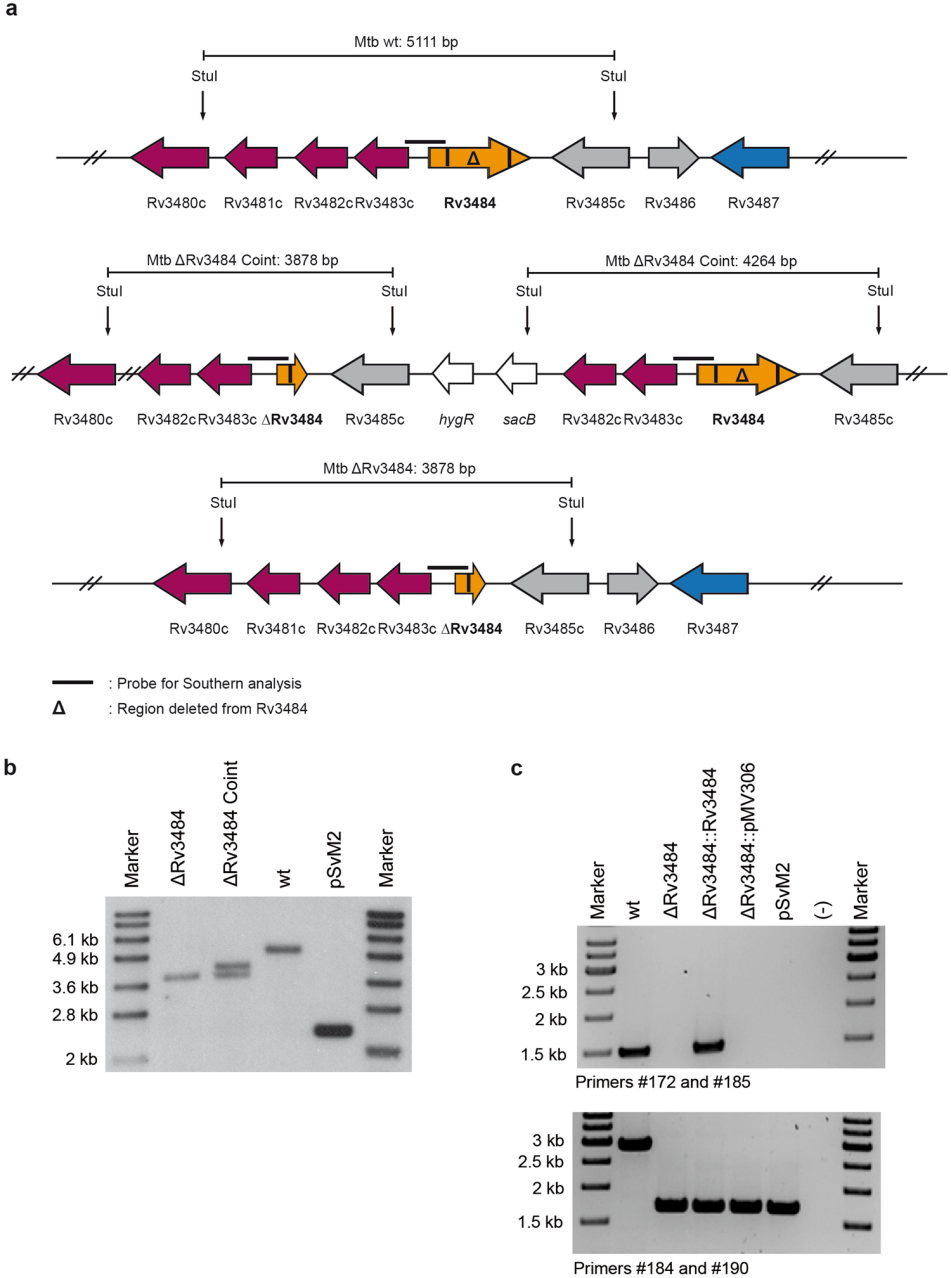
Genomic locus and deletion of Rv3484 from the genome of *Mtb* H37Rv. (**a**) Genomic organization of the Rv3484 locus including restriction sites and localization of the probe for Southern analysis and localization of primers for PCR analysis. The in frame deletion comprises 1233 bp. Southern blot of the *Mtb* H37Rv Rv3484 deletion mutant. (**b**) Southern analysis using StuI treated genomic DNA resulting in signals at 3878 bp for the mutant strain, two signals at 3878 bp and 4264 bp for the strain which underwent a single crossover resulting in cointegration of a deleted copy of Rv3484 and 5111 bp for the wild type. pSvM2 cut with PacI served as a control and resulted in a fragment of 2266 bp. PCR analysis of the *Mtb* H37Rv Rv3484 deletion mutant. (**c**) Agarose gels showing *Mtb* H37Rv wild type (wt), the strain carrying a deletion in Rv3484 (ΔRv3484) besides the complemented mutant strain (ΔRv3484::Rv3484), the mutant strain carrying the empty vector used for complementation (ΔRv3484::pMV306) and the plasmid used for transformation of the deleted copy of the gene (pSvM2) and a PCR negative control ((−)) as controls. Primers #172 and #185: wt 1481 bp, ΔRv3484::Rv3484 1481 bp. Primers #184 and #190: wt 2913 bp, ΔRv3484, ΔRv3484::Rv3484, ΔRv3484::pMV306, pSvM2: 1680 bp.

**Figure 2:**
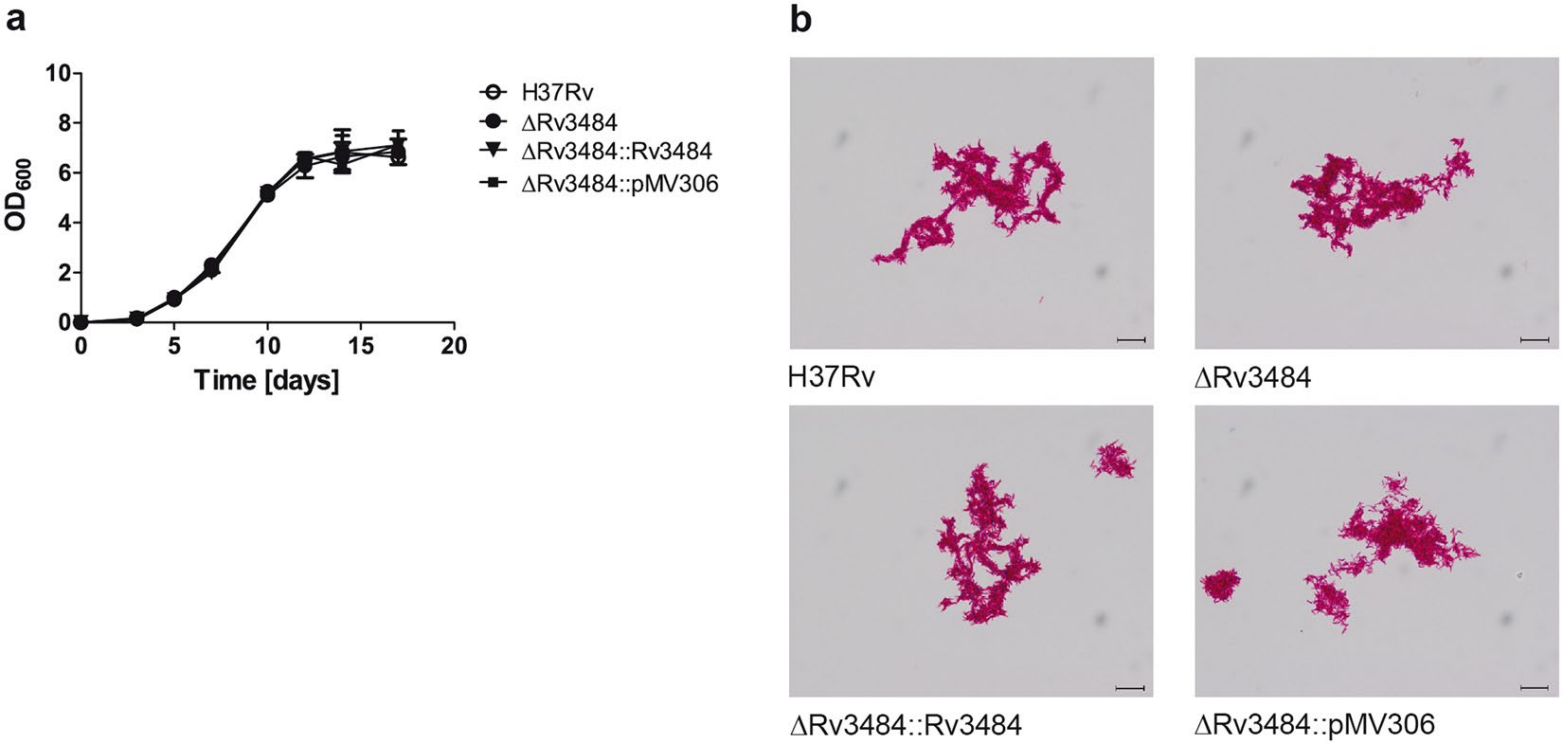
Growth kinetics of the Rv3484 deletion mutant strain in 7H9 medium and acid fast properties. (**a**) Bacteria were grown in fully supplemented 7H9 medium and OD_600_ measured at indicated time points. Means ± SD of biological triplicates performed in technical duplicates are shown. (**b**) Acid fast Ziehl-Neelsen staining of *Mtb* H37Rv, *Mtb* H37RvΔRv3484, *Mtb* H37RvΔRv3484::Rv3484 as well as H37RvΔRv3484::pMV306 was performed according to standard procedures (1000× magnification, scale ≙ 10 µm).

Rv3484 is a gene of 1539 bp encoding a 512 amino acids long protein. The majority (1233 bp) of Rv3484 was deleted in-frame using a two-step homologous recombination procedure resulting in a marker free mutant strain. Figure 1a shows the genomic locus of Rv3484 in *Mtb* H37Rv, the strain in which the plasmid providing the homologous recombination substrate had co-integrated into the chromosome, as well as *Mtb* H37RvΔRv3484, including restriction endonuclease sites used for Southern blotting, localization of the probe and expected fragment sizes. Inactivation of Rv3484 was confirmed by Southern blotting (Fig. 1b), PCR (Fig. 1c) and sequencing of the Rv3484 region (data not shown). Rv3484 is not essential *in vitro* as mutant clones could readily be obtained after the second intrachromosomal recombination event. *Mtb* H37RvΔRv3484 showed normal growth kinetics *in vitro* in 7H9 medium (Fig. 2a) and retained its acid-fast properties as shown by Ziehl-Neelsen staining (Fig. 2b).

### Resistance of Mtb H37RvΔRv3484 to *in vitro* stress conditions and hydrophobic properties

**Figure 3:**
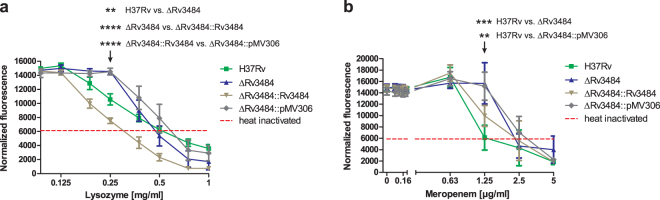
Resistance of the *Mtb* H37Rv Rv3484 deletion mutant against lysozyme. (**a**) *Mtb* wild type (H37Rv), *Mtb* H37RvΔRv3484, *Mtb* H37RvΔRv3484::Rv3484 as well as the mutant strain transformed with the empty vector pMV306 were exposed to lysozyme for 7 days and metabolic activity assessed using resazurin. Fluorescence was read at 590 nm in a microplate reader and the means and SEM of fluorescence intensities corrected by subtracting background fluorescence from wells containing dH_2_O and resazurin alone were plotted. Two-way ANOVA and Bonferroni Post-hoc-test were used for statistical evaluation of the data. P-values ≤ 0.01 (**) and ≤0.0001 (****) were considered as significant. (**b**) Resistance of the *Mtb* H37Rv Rv3484 deletion mutant strain against meropenem/clavulanate. *Mtb* wild type (H37Rv), *Mtb* H37RvΔRv3484, *Mtb* H37RvΔRv3484::Rv3484 as well as the mutant strain transformed with the empty vector pMV306 were exposed to 0, 0.04, 0.08, 0.16, 0.32, 0.63, 1.25, 2.5, 5 µg/ml meropenem (each sample supplemented with 2.5 µg/ml clavulanate) and metabolic activity of the bacteria was assessed after 1 week by the addition of resazurin. Data are expressed as normalized fluorescence intensities after subtraction of background fluorescence emitted by wells containing resazurin in 7H9 medium. Comparisons were based on two-way ANOVA and the Bonferroni Post-hoc-test. P-values ≤ 0.01 (**) or ≤0.001 (***) were considered as significant. Means with error bars representing the SEM are shown.

First, we addressed the question whether deletion of Rv3484 affects the resistance of *Mtb* H37RvΔRv3484 to certain stresses *in vitro*. To test the resistance to lysozyme, an enzyme cleaving the β-1,4-glycosidic bonds of the peptidoglycan backbone, we used the resazurin (7-hydroxy-10-oxidophenoxazin-10-ium-3-one) microplate assay. Redox dyes such as resazurin have been widely used to assess metabolic activity of viable cell lines^25^ as well as efficacy of antibiotics to bacterial pathogens including the *Mtbc*^26,27^. This assay measures the metabolic activity of the cells by means of resazurin reduction, yielding the fluorescent product resorufin. Results of this approach have been shown to correspond well with those obtained from traditional drug susceptibility testing^28–30^, and is recommended by the WHO for non-commercial drug susceptibility testing^31^. A lack of metabolic activity is regarded as indicative for an inhibitory activity of the compound tested but not necessarily its bactericidal activity. The resazurin microplate assay indicated that the deletion of Rv3484 resulted in an increased resistance to lysozyme (Fig. 3a). In the presence of 0.25 mg/ml lysozyme, we observed a significant decrease in fluorescent units of the *Mtb* H37Rv as compared to *Mtb* H37RvΔRv3484; in addition, differences were significant between the complemented mutant (*Mtb* H37RvΔRv3484::Rv3484) compared to *Mtb* H37RvΔRv3484 or the mutant strain transformed with the empty vector pMV306 (*Mtb* H37RvΔRv3484::pMV306).

In order to validate the experimental approach, we determined the OD_600_ of the samples prior to adding resazurin. Lysozyme was used as a stressor. The OD_600_ reads showed relatively high variation and less resolution than the fluorescence reads (Supplementary Fig. 1). Overall, the OD_600_ corresponded to the results obtained from the fluorescence reads as *Mtb* H37Rv and *Mtb* H37RvΔRv3484::Rv3484 tended to have the least OD_600_ reads when compared to *Mtb* H37RvΔRv3484 and *Mtb* H37RvΔRv3484::pMV306. Another way to read resazurin assays is to determine visually the concentration at which no metabolic activity could be detected making use of the change in color as a result of resazurin reduction. We detected a clear difference of one two-fold dilution between the pairs *Mtb* H37RvΔRv3484, *Mtb* H37RvΔRv3484::pMV306 and *Mtb* H37Rv, *Mtb* H37RvΔRv3484::Rv3484, respectively. We conclude that analysis of the fluorescence reads from the resazurin microplate assays is a suitable approach and allows for statistical analysis and assessment of observed effects of independent replicates.

Moreover, we analyzed whether osmotic stress (0.5 M NaCl) as an indicator for compromised cell wall homeostasis impacts *Mtb* H37RvΔRv3484 differently from *Mtb* H37Rv. However, we could not detect significant differences between mutant and the *Mtb* H37Rv regarding resistance against osmotic stress (Supplementary Fig. 2).

In addition, we tested the hydrophobicity of the bacteria by means of bacterial affinity to the hydrocarbon hexadecane in a biphasic system, but did not find differences between *Mtb* H37RvΔRv3484, *Mtb* H37Rv and *Mtb* H37RvΔRv3484::Rv3484 (Supplementary Fig. 2).

### Resistance of Mtb H37RvΔRv3484 to antibiotics

Since the mycobacterial cell wall is an important factor for the *Mtbc* to withstand antibiotic intervention, we tested the sensitivity of *Mtb* H37RvΔRv3484 to antibiotics. Bacteria of the *Mtbc* encode for a β-lactamase^32^; therefore, we added 2.5 µg/ml of the β-lactamase inhibitor clavulanate proven to render β-lactam antibiotics such as meropenem more effective^33^. Results obtained from the resazurin assay suggested that *Mtb* H37RvΔRv3484 is more resistant to meropenem/clavulanate than *Mtb* H37Rv or *Mtb* H37RvΔRv3484::Rv3484 (Fig. 3b).

For rifampicin we achieved ambiguous results (Supplementary Fig. 3). In addition, we did not observe differences in the susceptibilities to isoniazid, ethambutol, streptomycin, kanamycin, hygromycin and vancomycin between *Mtb* H37RvΔRv3484 and *Mtb* H37Rv (Supplementary Fig. 4). As expected, the control strains bearing the vector used for complementation or the empty vector were resistant to hygromycin as they carry a hygromycin resistance cassette as selection marker whereas no difference could be observed for *Mtb* H37RvΔRv3484 and *Mtb* H37Rv.

### Mtb H37RvΔRv3484 is severely attenuated in the mouse model of tuberculosis

**Figure 4:**
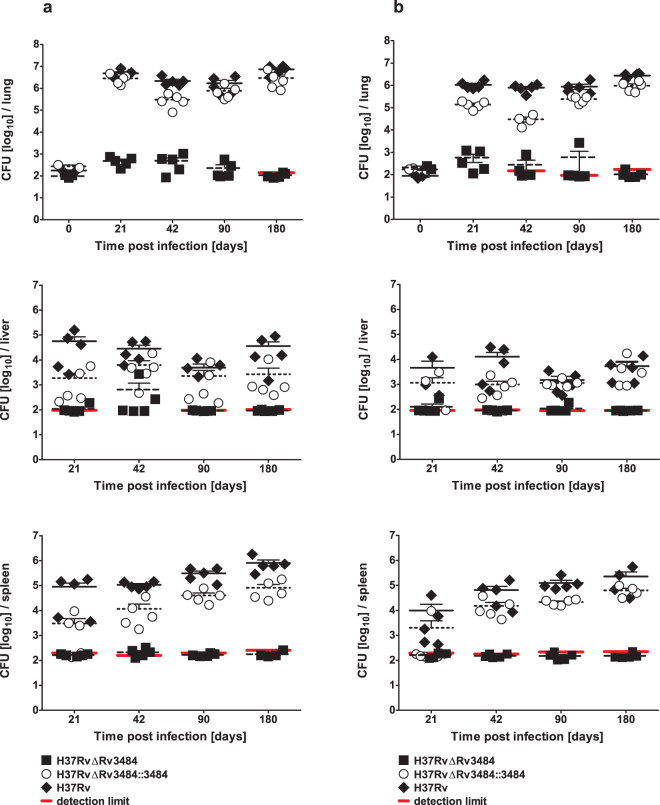
*In vivo* characterization of the Rv3484 deletion mutant of *Mtb* H37Rv. C57BL/6 mice were infected in two replicate experiments by aerosol containing either *Mtb* H37Rv ((**a**) 177 (**b**) 88 CFU/lung), *Mtb* H37Rv ΔRv3484 ((**a**) 98 (**b**) 210 CFU/lung) or *Mtb* H37Rv ΔRv3484::Rv3484 ((**a**) 274 (**b**) 171 CFU/lung) (infection dose determined with n = 2–3). At indicated time points the bacterial load of lung, spleen and liver was determined by enumerating bacteria after plating organ homogenates of 7H10 agar plates (n = 4–5 in each replicate experiment). A detection limit was calculated for samples for which at least for one animal no bacterial load could be detected by plating 100 µl of the organ homogenates. In this case raw CFU counts were set to 0.99, the theoretical CFU/organ calculated and defined as detection limit. Data points for organs of single mice as well as means ± SEM are shown.

In order to examine the role of Rv3484 as a virulence determinant, we infected C57BL/6 mice with an aerosol containing *Mtb* H37Rv, *Mtb* H37RvΔRv3484 and the complemented mutant strain *Mtb* H37RvΔRv3484::Rv3484 (infection dose approximately 100 CFU). Infections were performed twice with 5 mice per bacterial strain and time point. *Mtb* H37RvΔRv3484 was unable to establish an infection as compared to *Mtb* H37Rv and *Mtb* H37RvΔRv3484::Rv3484 (Fig. 4). The pulmonary bacterial loads of mice infected with *Mtb* H37RvΔRv3484 were similar or only slightly increased at day 21 to the infection dose assessed at day 1 indicating only weak, if any, replication in the early phase of infection (Fig. 4). Subsequently, the bacterial load declined and no bacteria could be detected 180 days *post infectionem* (*p.i*.). In comparison, both, the *Mtb* H37Rv and the complemented mutant strain grew with bacterial loads reaching 6–7 log units by day 21 *p.i*., which remained at a plateau until day 180 *p.i*. Despite the fact of extremely low bacterial loads in lungs of mice infected with *Mtb* H37RvΔRv3484, some bacteria were able to disseminate to liver and spleen. However, there was no evidence for replication. Single bacteria were detectable in some mice up to day 90. At day 180 *Mtb* H37RvΔRv3484 was cleared from all organs examined.

**Figure 5:**
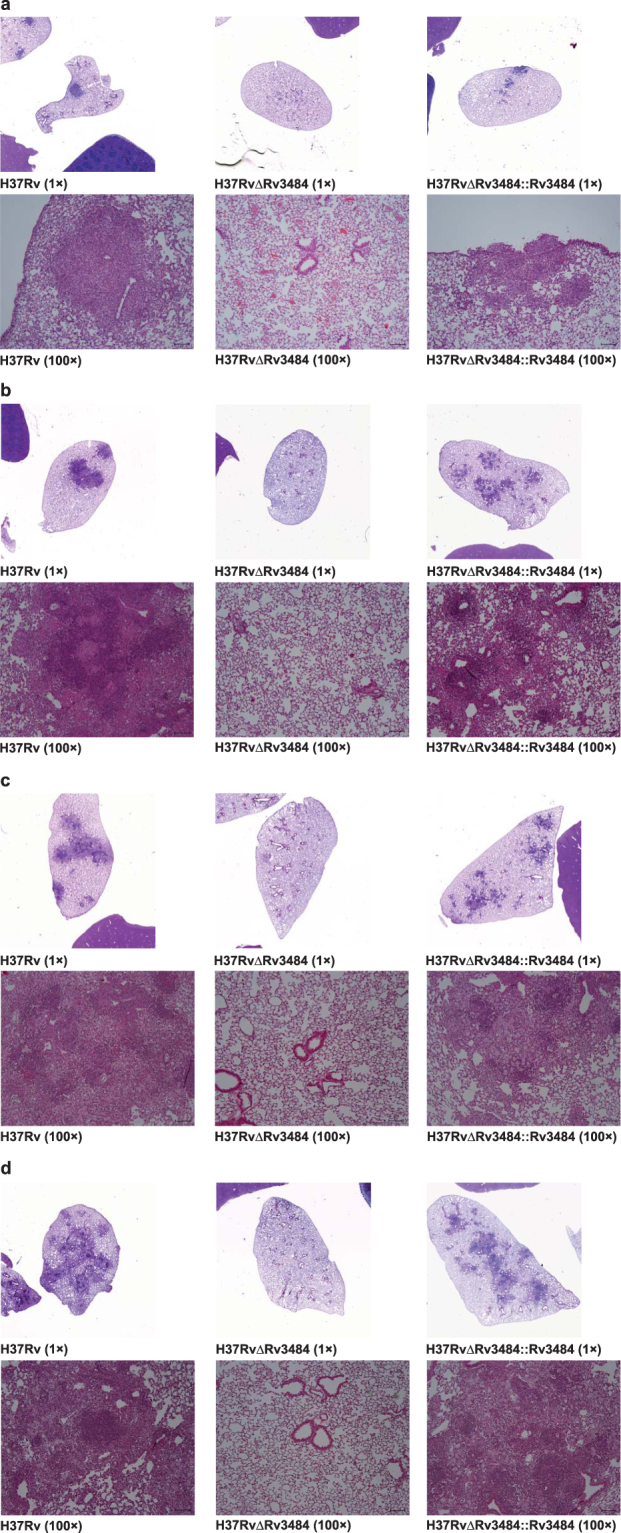
Histopathology of lung sections of mice infected with the *Mtb* H37Rv Rv3484 deletion mutant. HE stained sections of lungs prepared at day 21 (**a**), 42 (**b**), 90 (**c**) and 180 (**d**) post-infection scanned with 1× (original scan area 1 cm × 1 cm) using a slide scanner and 100 × (scale ≙ 100 µm) magnification of wild type (H37Rv; confirmed infection dose 88 CFU/lung), the Rv3484 deletion mutant (H37Rv∆Rv3484; confirmed infection dose 210 CFU/lung) and the complemented mutant strain (H37Rv∆Rv3484::Rv3484; confirmed infection dose 171 CFU/lung).

To assess whether the mutant strain was totally eliminated we plated 1 ml undiluted homogenate from mice infected for 180 days. Only very few colonies were recovered after 6 weeks of incubation (1, 1, 2, 5 and 10 CFU [5 animals out of 10; 3× lung, 1× liver, 1× spleen]). Four out of these five mice came from the replicate experiment, in which the infection dose for *Mtb* H37RvΔRv3484 was twice the one of *Mtb* H37Rv (210 versus 88 CFU/lung), which may be the reason for the slight delay in bacterial clearance. Strong attenuation of *Mtb* H37RvΔRv3484 is also reflected by the absence of inflammatory infiltrates in lungs from mice infected with *Mtb* H37RvΔRv3484 at all time points *p.i*. analyzed (Fig. 5). In contrast, granulomatous lesions were observed in lungs infected with *Mtb* H37Rv or *Mtb* H37RvΔRv3484::Rv3484 from day 21 on until day 180 *p.i*.

## Discussion

Our results demonstrate that Rv3484 is essential for growth and survival of *Mtb* H37Rv *in vivo*, in mice infected by aerosol. In contrast to *Mtb* H37Rv and *Mtb* H37RvΔRv3484::Rv3484, the marker free *Mtb* H37RvΔRv3484 was not able to establish a progressive infection in the aerosol mouse infection model.

Recently, Rv3484 has been proposed to encode for a LCP protein responsible for the transfer of AG to PGN. However, no major differences of the single mutant’s glycosyl compositions were detected, which was likely due to mutual functional compensation by these LCP proteins^23^. A detailed biochemical characterization using a conditional Rv3267/Rv3484 *Mtbc* double knockout strain in order to evaluate the proposed function is currently lacking. In addition, *in vivo* data are not available to date, and the relevance of this particular LCP protein homologue, out of the four LCP proteins found in *Mtb*, was yet studied. Indeed, functional redundancy between Rv3484 and Rv3267 was proposed. However, by establishing an unmarked Rv3484 deletion mutant, we were able to demonstrate an exclusive function of this LCP protein for *in vivo* virulence in mice.

Our own previous experiments showed that the expression of Rv3484 was upregulated universally in 17 representative strains of the *Mtbc* in response to conditions they encounter in mBMDMs^4^, indicating its importance for virulence of strains across distinct *Mtbc* lineages. Indeed, the *Mtbc* was proven to be more diverse than previously anticipated, which is presumably reflected in the successful spread of certain lineages in different areas of the world^34–36^. This resulted in particular host-pathogen-coevolution leading to differences in strain pathobiology as well as functional heterogeneity in *in vitro* and *in vivo* infection models; e.g. lineages of the *Mtbc* show specific virulence patterns in human primary macrophages and aerogenically infected C57BL/6 mice^37^. The conservation of putative cell wall synthesis associated domains in LCPs and the role of Rv3484 homologues in other bacteria^6–15^ together with its universal upregulation in response to macrophage infection in a diverse set of clinical *Mtbc* isolates^4^, prompted us to investigate the significance of this gene for *Mtbc* virulence in the reference strain, H37Rv. Indeed, our experiments showed, that the deletion mutant failed to establish infection in mice, thus, identifying Rv3484 as an essential gene for growth *in vivo*.

The mycobacterial cell wall is an important virulence determinant providing resistance to stresses *Mtbc* strains encounter during the course of infection, either simply by its rigidity, by masking pathogen associated molecular patterns e.g. by PDIM, or by immune modulatory compounds^38,39^. Defects in cell wall synthesis, especially disturbed homeostasis of the PGN backbone, can also lead to differential sensitivities to enzymes and antibiotics, which target this important structural component of the bacterial cell. Various antibiotics target PGN of bacterial cell walls. One example recently attracting much interest due to its mycobactericidal activity is meropenem, which shows increased efficacy in the presence of the β-lactamase inhibitor clavulanate^33^. We found increased resistance to meropenem/clavulanate of *Mtb* H37RvΔRv3484, while results for rifampicin were slightly ambiguous; however, *Mtb* H37RvΔRv3484 seemed to have increased resistance to this antibiotic, too. As rifampicin is not targeting the cell wall, increased resistance of the mutant strain could be due to reduced access of the drug to the mycobacterial cytoplasm to target the DNA-dependent RNA polymerase. Moreover, and in contrast to all the other antibiotics tested, to which both, mutant and wild-type strain, were equally susceptible, meropenem and rifampicin are both only sparingly soluble in water. However, analysis of *Mtb* H37RvΔRv3484 and *Mtb* H37Rv surface hydrophobicity using a biphasic hexadecane system did not reveal differences between both strains. Accordingly, differences in cell wall hydrophobicity that can determine permeability to compounds depending on their hydrophobic/hydrophilic properties cannot explain this observation. Grzegorzewicz *et al*. reported no differences between the Rv3484 mutant and wild type regarding the sensitivity to rifampicin. Slight differences in the experimental set-up, e.g. the usage of an auxotrophic H37Rv and the CDC1551 background, Tween 80 instead of Tyloxapol as a medium supplement, which was interchangeably used by Grzegorzewicz *et al*. for mutants derived from the two wild-type strains used in this work^23^, or other factors, might also influence the differential outcome. Taken together, we believe that rifampicin resistance, in contrast to the one against lysozyme and meropenem/clavulanate, does not represent a clear biological property which can characterize the Rv3484 deletion phenotype but rather a secondary effect.

The fact that Grzegorzewicz *et al*. did not observe a phenotype for the Rv3484 single KO, including no effects on the biochemical composition of the cell wall *in vitro*, let us refrain from thorough analytical chemistry approaches^23^. However, as we found a strong phenotype, which manifested predominantly *in vivo* we assume that the phenotype causing the attenuation might only be detectable in the *in vivo* situation e.g. directly from materials isolated from the lungs of infected mice at early time points or in response to stressors not yet identified. The experimental procedure and methodology to address this issue still need to be established. Furthermore, we cannot yet exclude the possibility that the *in vivo* attenuation we observed might be due to factors other than modified cell wall composition. Our results clearly show that Rv3484 function is essential for infection of mice. The phenotype, based on Rv3484 deficiency, becomes obvious already at early time points *p.i*. and results in almost complete elimination of the bacteria; thus, Rv3484 awaits further evaluation for its suitability as target for anti-TB treatment.

Only recently, another study supported the proposed function of the LCP homologues in *C. glutamicum* where biochemical analyses revealed a decreased amount of arabinogalactan attached to the cell wall in a mutant strain. Moreover, the importance of the C-terminal LytR_C domain was shown. Either concerted action of both domains on one substrate or subsequent cascade of two catalytic reactions has been surmised^40^. To answer whether the LytR_C domain plays a distinct role for *Mtb* survival *in vivo* requires additional genetic approaches.

In contrast to the impaired growth of the *M. marinum* mutant defective in the Rv3484 orthologue observed *in vitro*^22^, but in agreement with Grzegorzewicz *et al*.^23^, we found similar growth rates for *Mtb* H37Rv, *Mtb* H37Rv∆Rv3484, *Mtb* H37Rv∆Rv3484::Rv3484 and *Mtb* H37Rv∆Rv3484::pMV306. In contrast Rv3484 is essential for *Mtbc* survival in the TB mouse model, as only in some mice and organs very few bacteria of *Mtb* H37RvΔRv3484 could be detected in the lungs, spleen or liver of aerosol infected C57BL/6 mice. This severe attenuation of *Mtb* H37RvΔRv3484 could be complemented by the introduction of a single copy of Rv3484 under the control of its native promoter in the chromosome of *Mtb* H37RvΔRv3484 using an integrative vector. This finding sheds a different light on the role of this particular LCP protein Rv3484 and is in clear contrast to the functional redundancy proposed by Grzegorzewicz *et al*.^23^. The exact mechanism underlying our observation is currently addressed by structural and molecular biological approaches.

## Methods

### Bacterial strains and culture conditions

*Mycobacterium tuberculosis* H37Rv ATCC 27294 was used as a parental strain for genetic manipulations. *Escherichia coli* HB101 served as a host for molecular cloning procedures. Middlebrook 7H10 agar (Becton Dickinson, Franklin Lakes, USA) containing 0.5% glycerol (Applichem, Darmstadt, Germany), 10% OADC (Becton Dickinson, Franklin Lakes, USA), and 0.05% Tyloxapol (Sigma-Aldrich, St. Louis, USA) for 7H9 broth if required supplemented with 50 µg/ml hygromycin (Carl Roth, Karlsruhe, Germany) was used to cultivate *Mtb* strains, whereas Luria Bertani (LB) medium (Carl Roth, Karlsruhe, Germany) was used to cultivate *E. coli*. LB supplemented with Ampicillin (Carl Roth, Karlsruhe, Germany) was used as a selective medium for *E. coli*. For animal infection experiments *Mtb* stocks were prepared as follows. Bacteria were grown in 7H9 broth to mid-exponential growth phase and aliquots frozen at −80 °C. Titers of viable bacteria were determined after thawing by plating serial dilutions of the aliquots on 7H10 agar plates. Single cell suspensions were prepared by pushing the bacteria 10 times through a Microlance 3 26-gauge needle (Becton Dickinson, Franklin Lakes, USA). These suspensions were used for aerosol infection.

### Generation of the Rv3484 deletion mutant of Mtb H37Rv

Flanking regions of Rv3484 were amplified using the primer pair Rv3484_KO_fus1 5′ - cggtgtcgggtcggtggtggt – cagcccgatgagcaagatg −3′, Rv3484_KO_PacI3 5′ - gagtct - ttaattaa - cgtgctccattcaacagtc −3′ and the primer pair Rv3484_KO_fus2 5′ - acatcttgctcatcgggctg – accaccaccgacccgacac −3′, Rv3484_KO_PacI_4 5′ - agtatg - ttaattaa - cgtcgaacgtgaactgag −3′. The resulting fragments were fused in a third self-primed PCR reaction via their complementary 5 prime end extensions and the primers Rv3484_KO_PacI_3 and Rv3484_KO_PacI_4. This final PCR fragment was cloned via PacI into the mycobacterial suicide plasmid pYUB657^41^ (kindly provided by WR Jacobs) to obtain pSvM2 and used to transform *Mtb* H37Rv ATCC 27294. The mutant strain was generated using the two step allelic exchange methodology and successful deletion of Rv3484 from the genome was confirmed by PCR, Southern blotting and sequencing. The Rv3484 mutant was complemented using the integrative mycobacterial shuttle plasmid pMV306 providing a single wild-type copy of Rv3484^42^. Briefly, Rv3484 including 400 bp of its upstream region and 208 bp of its downstream region was amplified using HindIII-restriction site containing primers Rv3484_HindIII1 5′ - gagtctaagctt-cacacaggccaggaccac −3′ and Rv3484_HindIII2 5′ - gtatgaaagctt-gggtctgcacgccattac −3′ and cloned in pMV306 cut with HindIII. The resulting plasmid pSM14 was used to transform the Rv3484 deletion mutant of *Mtb* ATCC 27294. Southern blotting was performed according to standard procedures. The probe was amplified from genomic DNA using the primer pair #103 5′ - gtcaatctcgtcagacacctaac −3′ and #104 5′ – gcccgatgagcaagatg −3′ yielding a probe of 518 bp. PCR for validation of the strains used in this study was performed using primer pair #172 5′ - cctgccctggtcggtct −3′ and #185 5′ - gggtctgcacgccattac −3′ specific for the wild-type copy of Rv3484 as one primer binds in the deletion and the other one in the genome/the genome fragment cloned into the integrative vector resulting in the amplification of 1481 bp in case a wild-type copy is present. A second PCR was set up in order to validate the genomic situation at the Rv3484 locus using the primers #184 5′ - cacacaggccaggaccac −3′ and #190 5′ – gaacgtcgaacgtgaactgag −3′. These primers do not amplify from the integrative complementation vector as one primer does not bind in the construct and give a fragment of 2913 bp for the wild type and 1680 bp in case Rv3484 is deleted.

### Ziehl-Neelsen staining

Acid-fast staining was performed according to the Ziehl-Neelsen standard procedure. Briefly, bacteria were fixed on glass slides by heat fixation followed by staining with carbol-fuchsin, destaining with HCl–ethanol, washing with dH_2_O and counterstaining using methylene blue followed by a final wash step with dH_2_O.

### *In vitro* susceptibility testing of Mtb H37RvΔRv3484 to antibiotics and lysozyme

The resazurin assay was used to determine the susceptibility to antibiotics and certain stress conditions^43,44^. The strains were cultured in fully supplemented 7H9 medium until they reached the exponential growth phase. After adjusting bacterial suspensions to an OD_600_ of 0.3, inoculi were further diluted 1:20 before submitting 100 µl to the assay. Lysozyme or antibiotic dilutions (lysozyme 0, 0.094, 0.125, 0.188, 0.250, 0.375, 0.500, 0.750, 1 [mg/ml]; meropenem (+2.5 µg/ml clavulanate) 0, 0.04, 0.08, 0.16, 0.32, 0.63, 1.25, 2.5, 5 [µg/ml]; or as indicated in the supplementary information) were prepared in 100 µl volume in a 96-well microtiter plate (Corning, Corning, USA) and mixed with the bacterial suspensions. After 7 days of incubation at 37 °C 30 µl 0.01% (w/v) resazurin (Sigma-Aldrich, St. Louis, USA) was added to the wells. After further incubation at 37 °C o/n plates were read in a Biotek Synergy 2 plate reader using the Gen5 software (Biotek, Winooski, USA) and emission detected at 590 nm using an excitation wavelength of 540 nm or MICs were evaluated by eye and pictures were taken for documentation of negative results. Fluorescence reads were corrected for background fluorescence by subtracting values obtained from wells only containing dH_2_O (lysozyme) or medium (antibiotics) and resazurin for each plate. Data were plotted as background corrected (normalized) fluorescence reads. Experiments were performed in 5–11 independent replicates (lysozyme 5–9, meropenem/clavulanate 6, rifampicin 8–11) each designed in technical duplicates, in case no effect was observed at least 2 independent experiments in technical duplicates were performed and pictures of representative 96-well plates taken for documentation. Data were evaluated statistically using two-way ANOVA followed by the Bonferroni post-hoc test as indicated in the figure legends and p-values ≤ 0.05 (*), ≤0.01 (**), ≤0.001 (***) and ≤0.0001 (****) were considered as significant. GraphPad Prism software (GraphPad, La Jolla, USA) was used for plotting and statistical analysis of the data. Adobe Photoshop CS5 (Adobe Systems, San Jose, USA) was used to crop images which, if they were adjusted, only as whole pictures.

### *In vivo* infection experiments

10–12 week old female C57BL/6 mice (Charles River, Sulzfeld, Germany) were infected via aerosol (Glas-Col, Terre-Haute, Indiana, USA) with approximately 100 CFU of each *Mtb* strain. The actual infection dose was confirmed one day *p.i*. by plating lung homogenates on 7H10 agar plates supplemented with glycerol and 10% bovine serum (Biowest, Nuaillé, France). At further time points (21, 42, 90 and 180 days *p.i*.) lung, liver and spleen were removed and dilutions of homogenates plated on 7H10 agar plates to determine the bacterial load (2 lobes of the lungs, ca. ½ of liver and spleen) and to assess the pathology based on hematoxylin and eosin (HE) stained sections of fixed and paraffin embedded organs. The detection limit was defined for samples in which at least for one sample no CFU could be detected by plating 100 µl organ homogenate. In this case, raw CFU counts were set to 0.99, the theoretical CFU/organ calculated and defined as detection limit. The experiments were approved by the Ministry of Energy, Agriculture, the Environmental and Rural Areas of the state Schleswig-Holstein, Germany, and comply with the legal requirements of the German Animal Protection law.

## Electronic supplementary material

Supplementary information
