# Supplementary material for: In vivo virulence of Mycobacterium tuberculosis depends on a single homologue of the LytR-CpsA-Psr proteins
Source: Sci Rep. 2018 Mar 2;8:3936. doi: 10.1038/s41598-018-22012-6 (PMC5834633; doi:10.1038/s41598-018-22012-6)
Supplement: Supplementary file 1 — Supplementary information [file 41598_2018_22012_MOESM1_ESM.pdf]

Supplementary information

In vivo virulence of *Mycobacterium tuberculosis* depends on a single homologue of the LytR-CpsA-Psr proteins

Authors list

S. Malm[1*], S. Maaß[1], U.E. Schaible[2], S. Ehlers[3], S. Niemann[1,4]

Affiliations

[1]Molecular and Experimental Mycobacteriology, Priority Area Infections, Research Center Borstel – Leibniz Center for Medicine and Biosciences, 23845 Borstel, Germany

[2]Cellular Microbiology, Priority Area Infections, Research Center Borstel – Leibniz Center for Medicine and Biosciences, 23845 Borstel, Germany

[3]Molecular Inflammation Medicine, Priority Area Infections, Research Center Borstel – Leibniz Center for Medicine and Biosciences, 23845 Borstel, Germany

[4]German Center for Infection Research, Borstel Site, Borstel, Germany

*To whom correspondence should be addressed: smalm@fz-borstel.de.

Supplementary methods

Correlation of fluorescence reads, optical density and visual read out of the resazurin assay

The resazurin assay was performed as described in the material and methods section of the manuscript. Before adding resazurin OD_{600} of the samples was measured at day 7. Values obtained for fully supplemented medium alone were subtracted as blank. At day 8 fluorescence was measured; in addition, inhibitory lysozyme concentrations were assessed visually and pictures taken of the plates.

Sensitivity to NaCl and determination of hydrophobicity indices

NaCl was used to pose osmotic stress to the bacteria. Briefly, mutant and control strains were grown in fully supplemented 7H9 medium until mid-exponential growth phase, harvested and resuspended to OD_{600} 0.05. Samples were supplemented with 0.5 M NaCl and incubated for 0, 4 and 12 days and plated for CFU enumeration. Survival indices were calculated by dividing the counts obtained from samples with 0.5 M NaCl by the counts obtained from samples without stress at the indicated time points. Experiments were performed in independent duplicates.

The hydrophobicity indices were determined as follows, mainly as described by Stokes *et al.* [1]. Briefly, bacteria were cultivated until mid-exponential growth phase, harvested by centrifugation, washed with PUM buffer pH 7.1 and the OD_{600} was adjusted to 0.7. 1.5 ml of bacterial suspensions were mixed with different volumes of hexadecane (0, 200, 400 and 800 µl) and incubated for 8 min at 37°C. Suspensions were vortexed for 8 seconds followed by phase separation for 15 min at room temperature (ca. 20-22°C). The aqueous phase was transferred to a cuvette and the optical density measured at 400 nm. *Escherichia coli* K12 was used as a control. The index is regarded as a measure of hydrophobicity of the bacterial cells by means of removal of hydrophobic material from the aqueous phase and expressed as the

percentage of material remaining in the aqueous phase compared to the samples which were not mixed with hexadecane.

Evaluation of the sensitivity of the Rv3484 mutant strain to rifampicin

The resazurin assay was used to evaluate the sensitivity of the Rv3484 deletion mutant to rifampicin and was performed as described in the material and methods section of the manuscript. As the inter-assay variation using rifampicin in the resazurin assay appeared relatively high we calculated the ratios of the fluorescence of wells from the genetically modified organisms to the fluorescence emitted from wells containing the wild type H37Rv for each independent experiment after background correction of the fluorescence reads and plotted those data in addition to the background corrected fluorescence reads.

Extended antibiotic susceptibility testing

In addition to meropenem/clavulanate and rifampicin we tested several other antibiotics in two-fold dilution series such as isoniazid (range 1.25 - 0.005 µg/ml), ethambutol (range 20 - 0.078 µg/ml), streptomycin (range 10 - 0.039 µg/ml), kanamycin (range 50 - 0.195 µg/ml), hygromycin (range 100 – 0.39 µg/ml) and vancomycin (range 400 – 1.56 µg/ml) using the resazurin assay as described in detail in the materials and methods section of the manuscript. Experiments were performed in two independent replicates with technical duplicates. Results were documented by taking pictures of representative 96-well assay plates.

References

1. Stokes, R. W. *et al.* The Glycan-Rich Outer Layer of the Cell Wall of Mycobacterium tuberculosis Acts as an Antiphagocytic Capsule Limiting the Association of the Bacterium with Macrophages. *Infect. Immun.* **72,** 5676–5686 (2004).

Supplementary figures

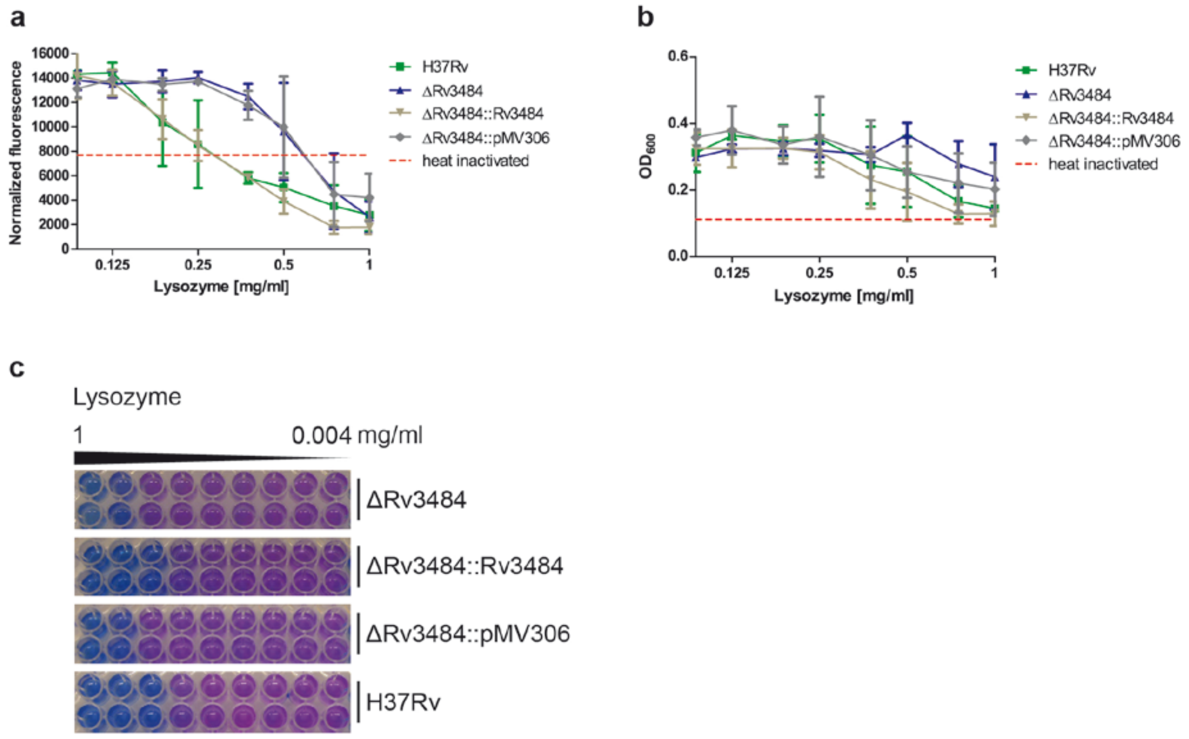

Supplementary figure 1. Validation of the resazurin assay. (**a**) Normalized fluorescence reads, (**b**) OD_{600} of samples read before addition of resazurin and (**c**) pictures taken of representative plates from the same experiments as shown in (**a**) and (**b**). Experiments for validation of the assay were performed in two biological replicates each in two technical duplicates. (**a**) and (**b**), means ± SEM are shown.

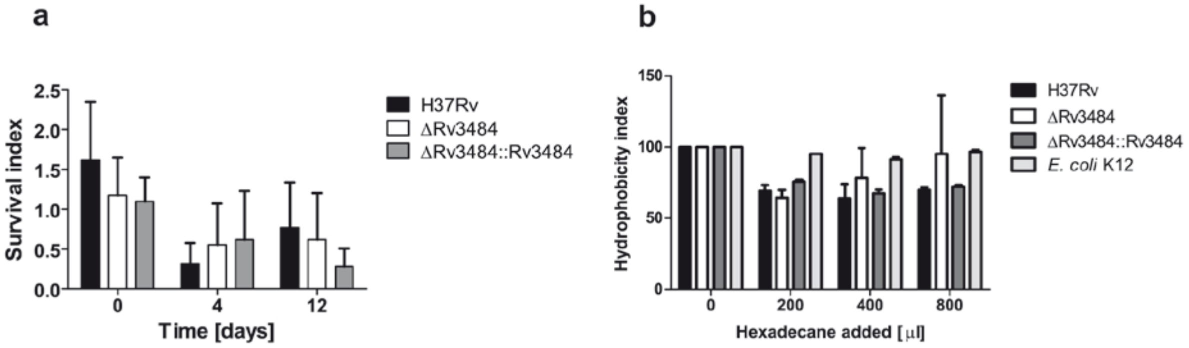

Supplementary figure 2. Resistance of the *Mtb* H37Rv Rv3484 deletion mutant to osmotic stress (NaCl) and hydrophobic properties. (**a**) Bacteria were grown to mid exponential growth phase, OD_{600} adjusted to 0.05 and then exposed to 0.5 M NaCl in 7H9 medium, incubated for 0, 4, 7 and 12 days and CFU determined by plating suspensions on fully supplemented 7H10 agar plates. Experiments were performed in two independent replicates, means and SEM calculated and data plotted as survival compared to bacteria grown without addition of NaCl. (**b**) The ability to form emulsions with the hydrocarbon hexadecane was utilized to assess the hydrophobic properties of the bacteria. Bacteria were mixed with different volumes of hexadecane and the OD_{400} of the aqueous phase measured thereafter. The indices represent the remaining OD_{400} in the aqueous phase as compared to samples without addition of hexadecane. Means with SEM of two independent experiments performed in technical duplicates are shown.

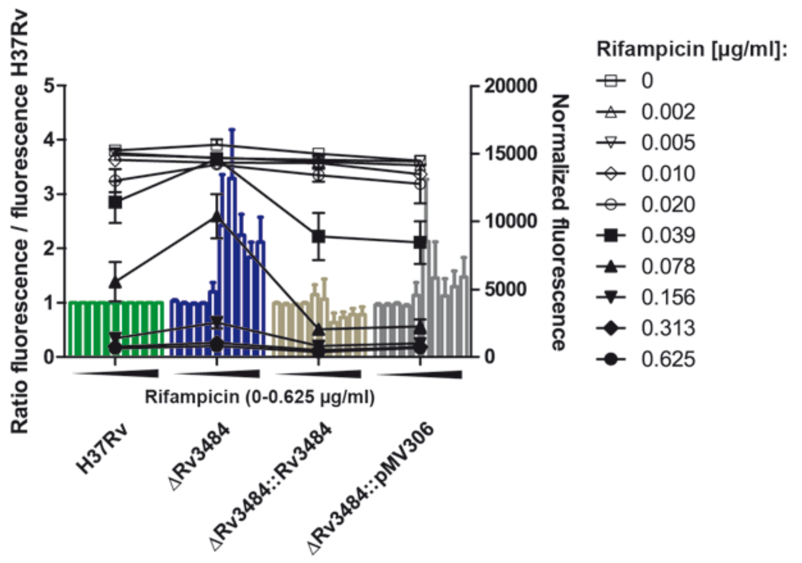

Supplementary figure 3. Behavior of the mutant strain in 7H9 medium supplemented with rifampicin. We calculated the ratios of the fluorescence of wells from the genetically modified organisms to the fluorescence emitted from wells containing the wild type H37Rv for each independent experiment (left y-axis, colored bars) after background correction of the fluorescence reads. Means and SEM are shown. In addition we plotted the means of background corrected fluorescence reads (right y-axis, black solid lines). Two-way ANOVA followed by Bonferroni Post-hoc-test was used to evaluate the data (Normalized fluorescence reads: H37Rv vs ΔRv3484: 0.039 µg/ml (*), 0.078 µg/ml (****); H37Rv vs ΔRv3484::Rv3484: 0.078 µg/ml (**); ΔRv3484 vs ΔRv3484::Rv3484: 0.039 µg/ml (****), 0.078 µg/ml (****); ΔRv3484 vs ΔRv3484::pMV306: 0.039 µg/ml (****), 0.078 µg/ml (****), comparison ΔRv3484, ΔRv3484::Rv3484 and ΔRv3484::pMV306 ratios: ΔRv3484 vs ΔRv3484::Rv3484: 0.078 µg/ml (****), ΔRv3484 vs ΔRv3484::pMV306 0.078 µg/ml (*)). Data were generated in 8-11 independent experiments in technical replicates. (*) $p \leq 0.05$, (**) $p \leq 0.01$, (****) $p \leq 0.0001$.

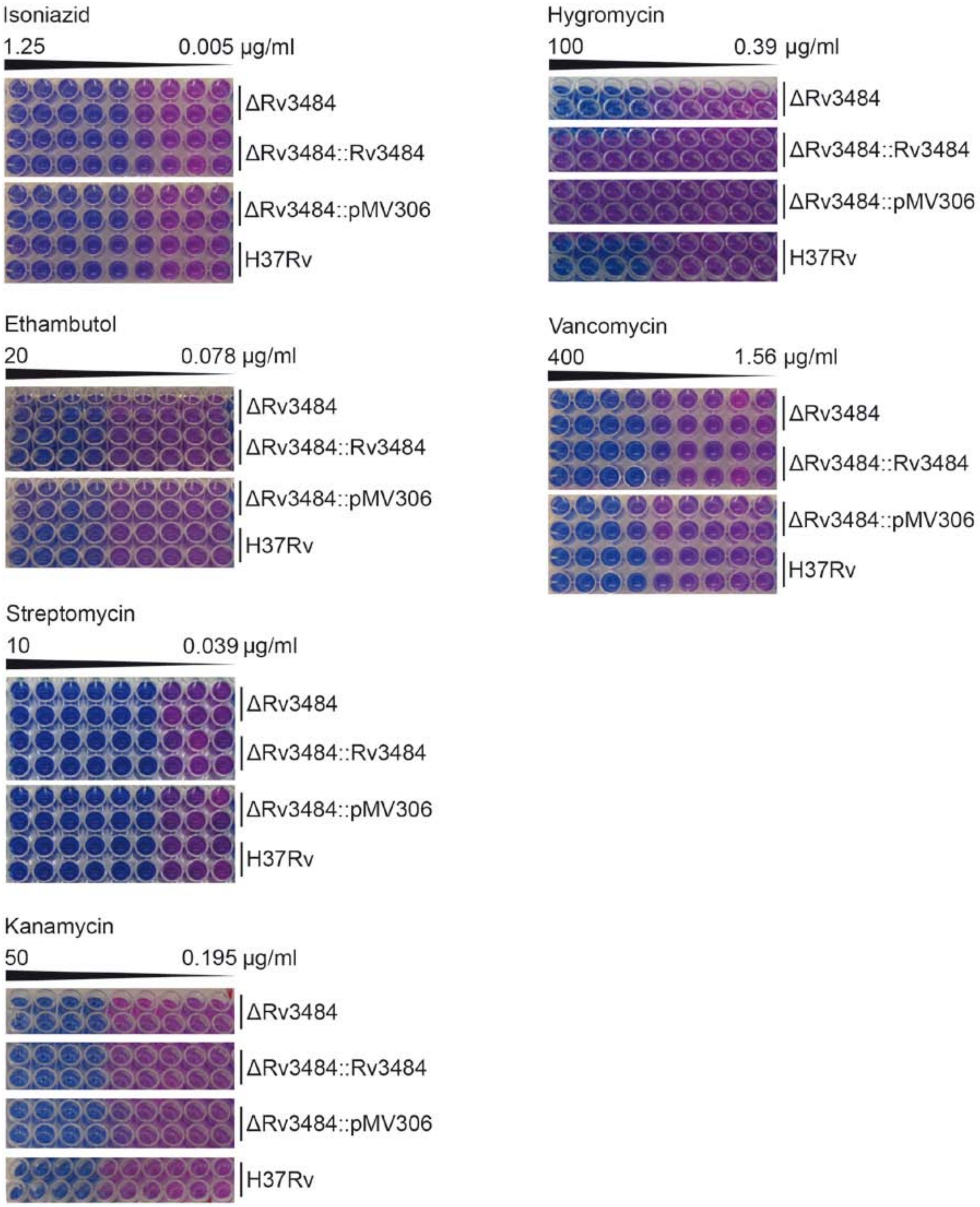

Supplementary figure 4. Additional testing of antibiotic susceptibility. *Mtb* wild type (H37Rv), *Mtb* H37RvΔRv3484, *Mtb* H37RvΔRv3484::Rv3484 as well as the mutant strain transformed with the empty vector pMV306 were exposed to two-fold dilution series of antibiotics as indicated in the figure and metabolic activity assessed using resazurin after 7 days of incubation. Two independent experiments were performed and pictures taken of representative microplates for documentation of negative results.
